# Suppression of Tumor Growth, Metastasis, and Signaling Pathways by Reducing FOXM1 Activity in Triple Negative Breast Cancer

**DOI:** 10.3390/cancers12092677

**Published:** 2020-09-19

**Authors:** Parama Dey, Alexander Wang, Yvonne Ziegler, Sung Hoon Kim, Dorraya El-Ashry, John A. Katzenellenbogen, Benita S. Katzenellenbogen

**Affiliations:** 1 Department of Molecular and Integrative Physiology, University of Illinois at Urbana-Champaign, Urbana, IL 61801, USA; paramad@illinois.edu (P.D.); awang65@illinois.edu (A.W.); yziegler@illinois.edu (Y.Z.); 2 Department of Chemistry, University of Illinois at Urbana-Champaign, Urbana, IL 61801, USA; kimsh@illinois.edu (S.H.K.); jkatzene@illinois.edu (J.A.K.); 3 Department of Laboratory Medicine and Pathology, University of Minnesota Medical School, Minneapolis, MN 55455, USA; delashry@umn.edu; 4 Cancer Center at Illinois, University of Illinois at Urbana-Champaign, Urbana, IL 61801, USA; 5 Carl Woese Institute for Genomic Biology, University of Illinois at Urbana-Champaign, Urbana, IL 61801, USA

**Keywords:** triple negative breast cancer, migration, invasion, metastasis, FOXM1, EMT

## Abstract

**Simple Summary:**

Triple negative breast cancer is an aggressive subtype of breast cancer that frequently metastasizes. Because the transcription factor FOXM1 is highly upregulated in triple negative breast cancer and controls many cell activities that lead to cancer progression and metastasis, we sought to determine if FOXM1 inhibitory compounds could effectively suppress the invasiveness and progression of triple negative breast cancer cells and tumors. Our findings show that these compounds inhibit cell motility, invasiveness, and the expression of important proteins associated with epithelial to mesenchymal transition. These compounds also suppressed the proliferation and metastatic outgrowth of triple negative breast tumors. Thus, these findings highlight the crucial role of FOXM1 in promoting the progression and metastasis of these cancers, and suggest that FOXM1 inhibitory compounds may have therapeutic potential and prove beneficial in intervention against triple negative breast cancer.

**Abstract:**

Metastasis-related complications account for the overwhelming majority of breast cancer mortalities. Triple negative breast cancer (TNBC), the most aggressive breast cancer subtype, has a high propensity to metastasize to distant organs, leading to poor patient survival. The forkhead transcription factor, FOXM1, is especially upregulated and overexpressed in TNBC and is known to regulate multiple signaling pathways that control many key cancer properties, including proliferation, invasiveness, stem cell renewal, and therapy resistance, making FOXM1 a critical therapeutic target for TNBC. In this study, we test the effectiveness of a novel class of 1,1-diarylethylene FOXM1 inhibitory compounds in suppressing TNBC cell migration, invasion, and metastasis using in vitro cell culture and in vivo tumor models. We show that these compounds inhibit the motility and invasiveness of TNBC MDA-MB-231 and DT28 cells, along with reducing the expression of important epithelial to mesenchymal transition (EMT) associated genes. Further, orthotopic tumor studies in NOD-SCID-gamma (NSG) mice demonstrate that these compounds reduce FOXM1 expression and suppress TNBC tumor growth as well as distant metastasis. Gene expression and protein analyses confirm the decreased levels of EMT factors and FOXM1-regulated target genes in tumors and metastatic lesions in the inhibitor-treated animals. The findings suggest that these FOXM1 suppressive compounds may have therapeutic potential in treating triple negative breast cancer, with the aim of reducing tumor progression and metastatic outgrowth.

## 1. Introduction

Triple negative breast cancers (TNBCs) represent a highly aggressive breast cancer subtype that very frequently develops treatment resistance and metastasizes to distant organs. This recurrence and metastatic spread is a leading cause of morbidity and mortality in patients with these breast cancers [1,2]. The transcription factor FOXM1 is overexpressed in breast cancers of all subtypes, but is especially high in TNBC, as well as in many other types of cancers, including ovarian, pancreatic, prostate, and glioblastoma [3,4,5,6,7,8,9,10,11]. Because FOXM1 upregulation to high levels is particularly common in TNBC, we have examined the possibility of reducing TNBC aggressiveness and metastasis by inhibiting FOXM1 activity. We recently identified and characterized novel FOXM1 inhibitory compounds, and demonstrated their beneficial role in suppressing cancer cell proliferation and tumor growth through cell cycle blockade and the induction of apoptosis [12].

While being a key cell cycle regulator, FOXM1 also plays a multifaceted role in cancer progression, stimulating cancer cell migration, invasion, angiogenesis, stem cell self-renewal and therapeutic resistance [4,9,13,14,15]. Additionally, FOXM1 transcriptionally regulates the expression of key players in epithelial to mesenchymal transition (EMT). These factors, including Snail, Slug, Vimentin, and MMPs, are directly involved in the early stages of cancer metastasis by allowing the conversion of adhesive epithelial cells to a more migratory phenotype, along with a change in the stromal compartment, thereby facilitating cell spread [16,17,18,19]. Therefore, targeting FOXM1 could prove to be a useful new therapeutic strategy, allowing the suppression of multiple key cancer regulatory pathways.

Our recent study established the antitumor efficacy of 1,1-diarylethylene mono and diamines, and their corresponding methiodide salts [12], which were shown to be highly efficacious in reducing expression of FOXM1 and its cell cycle targets, and in suppressing cancer cell proliferation and inducing apoptosis. Here, we have now tested the ability of FOXM1 inhibitory compounds to suppress TNBC progression and tumor metastasis. We observed a significant decrease in motility and invasiveness of TNBC cells when treated with these compounds, and our in vivo studies reveal that the FOXM1 inhibitory compounds attenuate tumor growth and distant metastasis, effects that were accompanied by reductions in the level of FOXM1 target genes and important players in epithelial to mesenchymal transition (EMT). These observations highlight the crucial role of FOXM1 in promoting the progression and metastasis of TNBC, and suggest that these compounds may hold promise for therapeutic intervention against TNBC.

## 2. Results

### 2.1. FOXM1 Inhibitors Reduce the Motility and Invasive Potential of Triple Negative Breast Cancer Cells

**Figure 1:**
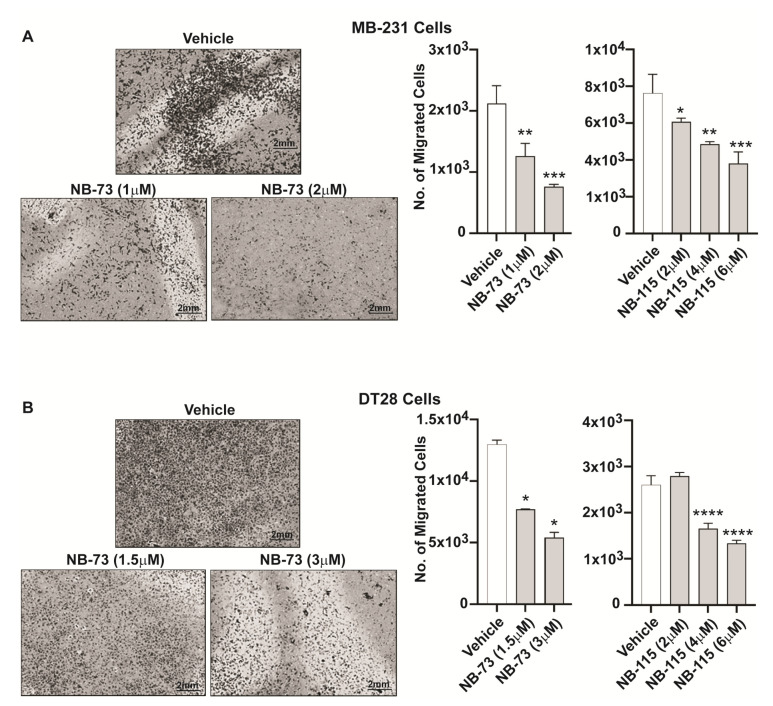
Dose-dependent effect of FOXM1 inhibitory compounds on migration of triple negative breast cancer (TNBC) cells, (**A**) MDA-MB-231 and (**B**) DT28 cells were treated with different concentrations of NB-73 and NB-115 for 48h, and the Boyden Chamber migration assay was conducted. At left, a few representative images of cell fields viewed microscopically are shown. Statistical analysis of data shown at right used one-way ANOVA and * *p* < 0.05, ** *p* < 0.01, *** *p* < 0.001 and **** *p* < 0.0001 are indicated; mean ± SD.

**Figure 2:**
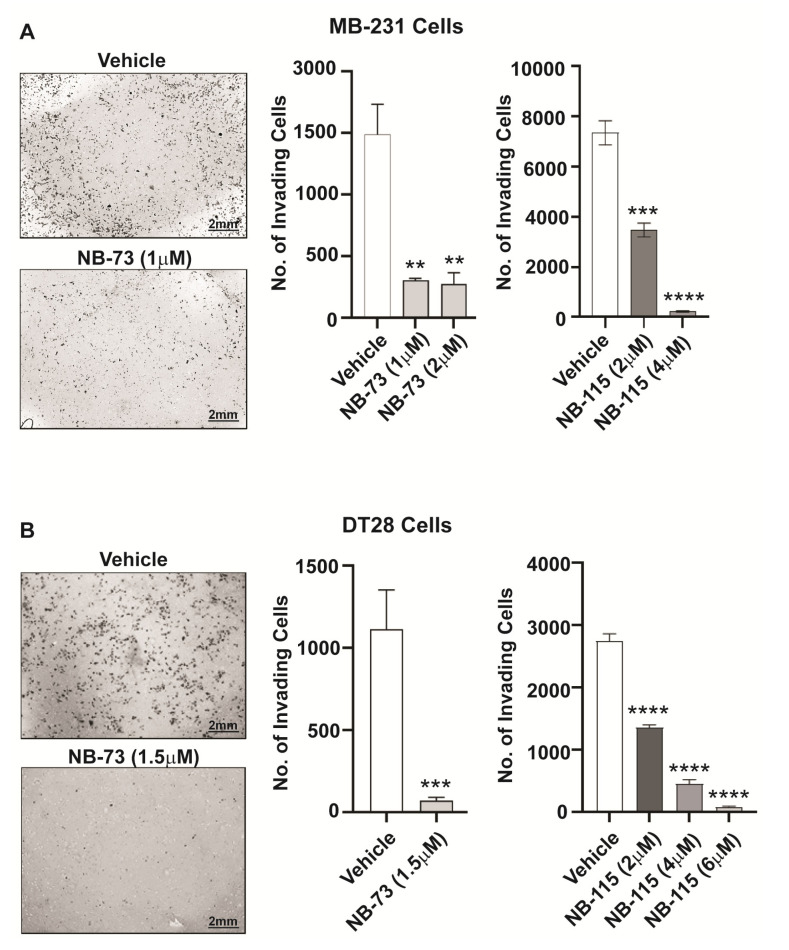
FOXM1 inhibitory compounds reduce invasive potential of TNBC cells, (**A**) MDA-MB-231 cells and (**B**) DT28 cells were treated for 48h with NB-73 or NB-115 and were monitored for invasion using Matrigel-coated cell culture inserts in 24-well plates. At left, a few representative images of cell fields viewed microscopically are shown. Statistical analysis of data shown at right used one-way ANOVA and * *p* < 0.05, ** *p* < 0.01, *** *p* < 0.001 and **** *p* < 0.0001 are indicated; mean ± SD.

As a prelude to examining the role of FOXM1 in TNBC metastasis, we first investigated the effect of our FOXM1 inhibitory compounds on the migration of TNBC cells. We observed that treatment with different concentrations of the compounds NB-73 and NB-115 resulted in a significant reduction in the motility of both MDA-MB-231 cells (Figure 1A) and DT28 cells (Figure 1B). In addition, because invasive properties of metastatic cancer cells are a necessary requirement for them to enter the bloodstream and facilitate transport to and lodging at distant organs, we also examined the effect of these FOXM1 inhibitory compounds on restricting cancer cell invasiveness. Following treatment with NB-73 or NB-115, a marked decrease in invasiveness of both MB-231 cells (Figure 2A) and DT28 cells (Figure 2B) was observed when the TNBC cells were seeded onto Matrigel-coated cell culture inserts in the top chamber and serum was used as a chemoattractant in the bottom chamber. In addition to these studies with the more potent compounds, NB-73 and NB-115, we also carried out some classical scratch migration assays with NB-73 and the less potent NB-55 compound, because the latter inhibitor is orally active and is used in later in vivo tumor suppression studies. As shown in Supplementary Figure S1A, NB-73 and NB-55 both reduced cell migration in the scratch assay, and they also reduced the expression of FOXM1 and FOXM1 target genes (Supplementary Figure S1B).

### 2.2. FOXM1 Inhibitors Downregulate EMT Marker-Associated Gene Expression

**Figure 3:**
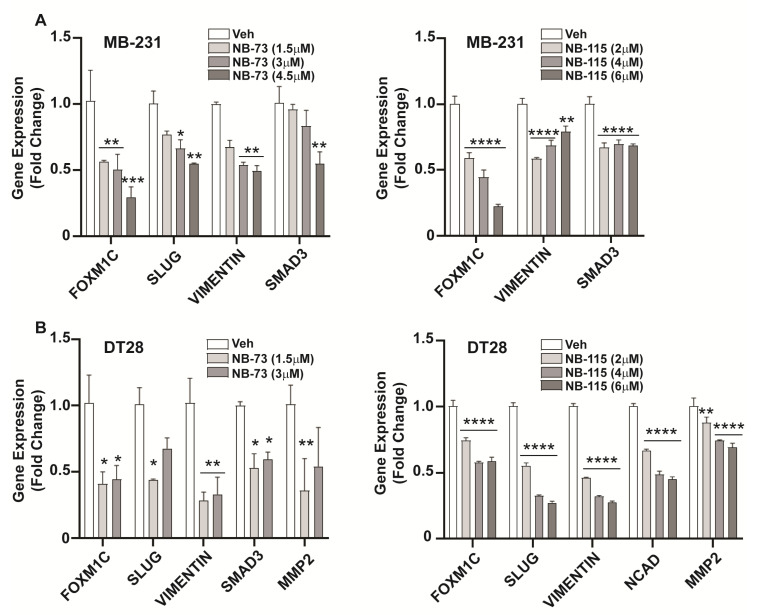
Downregulation of EMT-associated gene expression in inhibitor-treated TNBC cells, (**A**) MDA-MB 231 and (**B**) DT28 cells were treated for 24h with various concentrations of NB-73 and NB-115 followed by RNA extraction and qPCR analysis of EMT related gene expressions. Statistical analysis used 2-way ANOVA and * *p* < 0.05, ** *p* < 0.01, *** *p* < 0.001 and **** *p* < 0.0001 are indicated; mean ± SEM.

**Figure 4:**
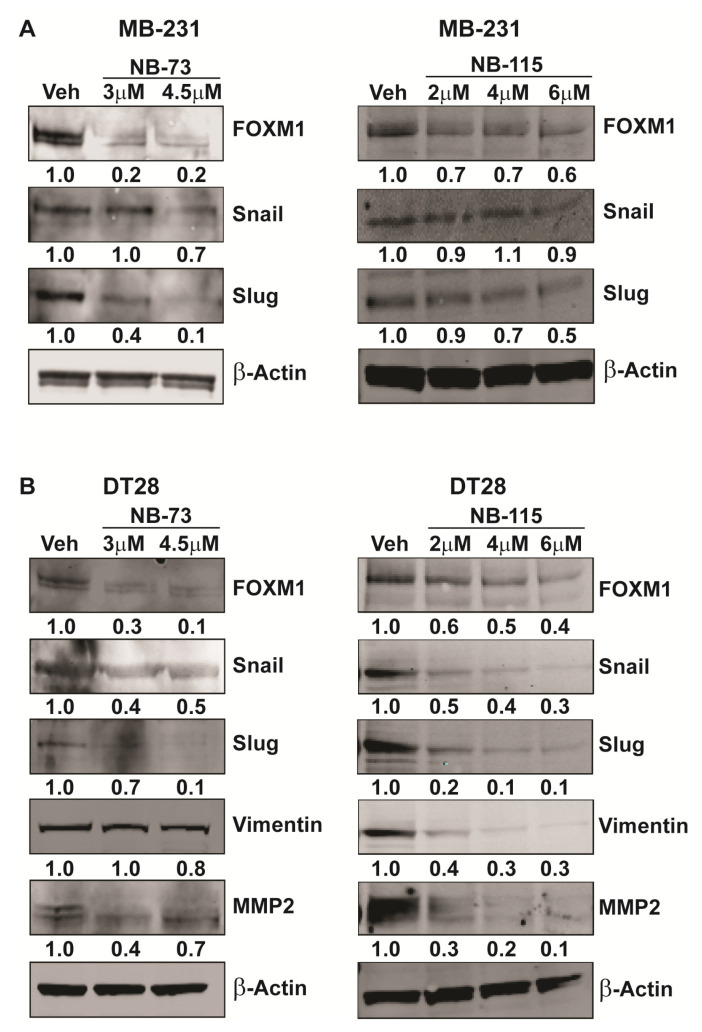
FOXM1 inhibition leads to decreased cellular levels of EMT proteins, Treatment of (**A**) MDA-MB-231 and (**B**) DT28 cells with NB-73 and NB-115 at the concentrations indicated for 24 h and subsequent Western blot analysis for FOXM1 and EMT marker proteins. β-Actin is used as a loading control. Full gel blots are shown in Supplementary Figures S3 and S4.

The process of metastasis during cancer progression involves the critical step of Epithelial to Mesenchymal Transition (EMT), wherein the epithelial cells acquire mesenchymal properties that confer increased motility and invasiveness. Having observed the effect of the FOXM1 inhibitory compounds on TNBC cell motility and invasion, we next investigated their effect on expression of important EMT-related genes known to be involved in breast cancer metastasis. Treatment of MB-231 or DT28 TNBC cells with NB-73 and NB-115 resulted in downregulation of the expression of EMT marker genes including Slug, Vimentin, SMAD3, and MMP2 (Figure 3A,B). Further, we observed dose-dependent decreases in the level of these EMT proteins in Western blots of NB-73 and NB-115 treated MB-231 and DT28 cells (Figure 4A,B; with the antibody dilution information in Supplementary Table S1 and full gel blots in Supplementary Figures S3 and S4). Therefore, treatment with these compounds resulted in reduced cellular levels of proteins associated with an important step in breast cancer metastasis. 

### 2.3. Effect of NB-73 and NB-115 on TNBC Tumor Growth and Metastasis

**Figure 5:**
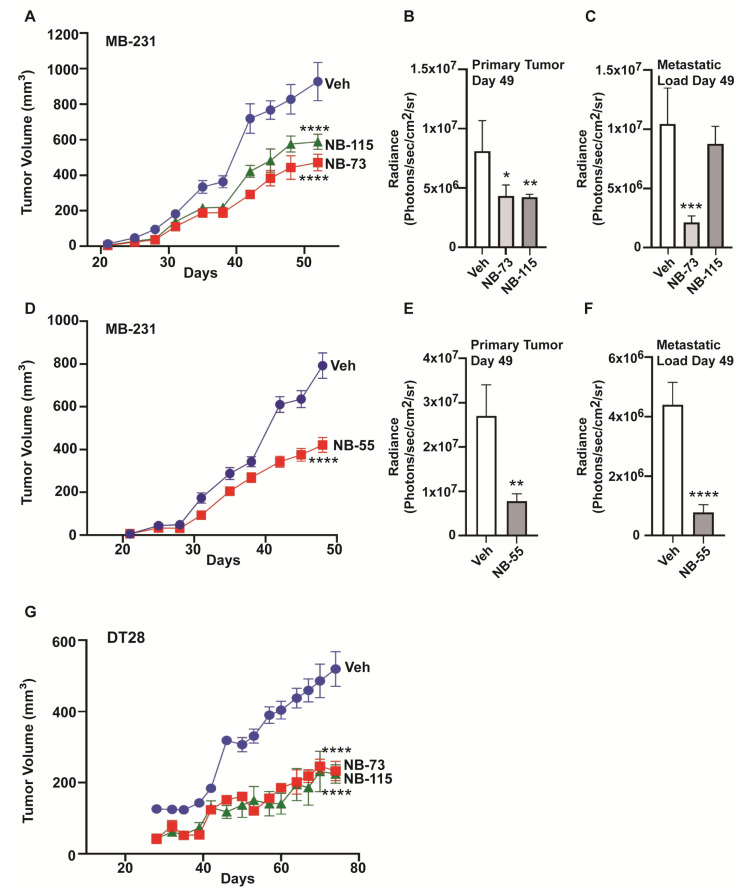
Compounds NB-73, NB-115, and NB-55 suppress TNBC tumor growth and metastasis in vivo, (**A**) Luciferase-expressing MDA-MB-231 cells were inoculated into the mammary fat pad of intact 7 week old NSG mice and mice bearing MDA-MB-231 tumors were dosed with 10 mg/kg of NB-73 or NB-115 or control vehicle by subcutaneous injection as detailed in Methods. Tumor volumes were monitored by caliper measurement of the primary tumors (2-way ANOVA, Dunnett’s post-test, ****, *p* < 0.0001). (**B**) Tumor growth was also monitored by IVIS bioluminescence at day 49. (**C**) Metastatic load in each group was also monitored by IVIS bioluminescence at day 49. (2-way ANOVA, *, *p* < 0.05; **, *p* < 0.01; ***, *p* < 0.001; ****, *p* < 0.0001; mean ± SEM). (**D**) Effect of NB-55 on MB-231 tumor growth and metastasis. NB-55 (100 mg/kg) or control vehicle was delivered by oral gavage as described in Methods. Tumor volume was measured using calipers (Multiple *t*-test, ****, *p* < 0.0001). (**E**) Tumor growth was also monitored by IVIS bioluminescence at day 49. (Multiple *t*-test, Sidak method, **, *p* < 0.01; mean ± SEM). (**F**) The metastatic load in mice was monitored by IVIS bioluminescence at day 49 (2-way ANOVA; ****, *p* < 0.0001; mean ± SEM). Bioluminescence is shown as Average Radiance per mouse (Photons/sec/cm2/sr). (**G**) Growth of luciferase-expressing DT28 xenograft tumors in animals receiving vehicle, NB-73, or NB-115, as in Panel A. 2-Way ANOVA multiple comparisons; **** *p* < 0.0001. Note that the *X*-axis scale (time in days) is different in this panel from that in panels A and D.

To further explore the effect of these compounds on breast tumor progression and metastasis in vivo, NOD-SCID-gamma (NSG) female mice were implanted with luciferase-positive MB-231 cells in the fourth left inguinal mammary gland, followed by sc treatment with NB-73 and NB-115, or oral treatment with NB-55, and tumor growth and distant metastasis were monitored. We observed a significant reduction in primary tumor growth in NB-73, NB-115, and NB-55 treated mice by measurement of tumor volume with calipers, as well as by IVIS bioluminescence imaging (Figure 5A,B,D,E). Metastatic load at distant sites, such as the lungs, was also significantly lower in NB-73 and NB-55 treated mice (Figure 5C,F) but not in NB-115 treated animals, perhaps reflecting the somewhat lower potency of this compound observed also in migration and invasion assays, where higher concentrations of NB-115 vs. NB-73 were needed. In the TNBC cell line, DT28, NB-73, and NB-115 also greatly reduced tumor growth (Figure 5G), but these tumors were smaller and grew more slowly than the MDA-MB-231 tumors, and we did not detect any metastases in mice with DT28 breast tumors over the 70 days of the study, in either control vehicle or compound-treated mice by IVIS bioluminescence imaging, or by examination of lung tissue. Likewise, q-PCR analysis failed to detect any luciferase expression in the lungs, indicating few, if any, DT28 cancer cells in the lung. Monitoring of animal weights over the course of the studies showed that weights increased or remained relatively constant over time, with animals showing some weight loss towards the end of the study, probably due to increased tumor and/or metastatic burden (Supplementary Figure S2).

### 2.4. Gene Expression Analyses in FOXM1 Inhibitor-Treated Tumors and Metastatic Lesions

**Figure 6:**
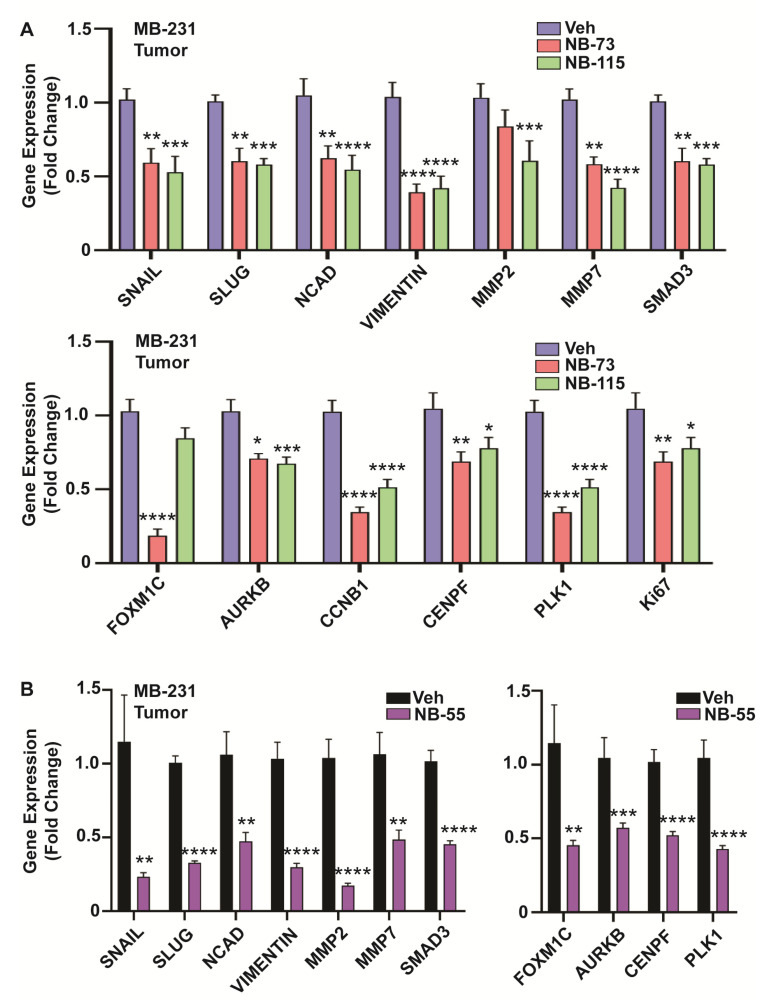
Gene expression analyses in FOXM1 inhibitor-treated primary TNBC tumors, MDA-MB-231 tumors were collected from all animals at the end of the studies, followed by RNA isolation and qPCR analysis for EMT markers and cell cycle targets of FOXM1. Treatment with (**A**) NB-73 and NB-115, administered subcutaneously and (**B**) NB-55, administered orally. (2-way ANOVA, Dunnett’s post-test, * *p* < 0.05, ** *p* < 0.01, *** *p* < 0.001, **** *p* < 0.0001; mean ± SEM).

**Figure 7:**
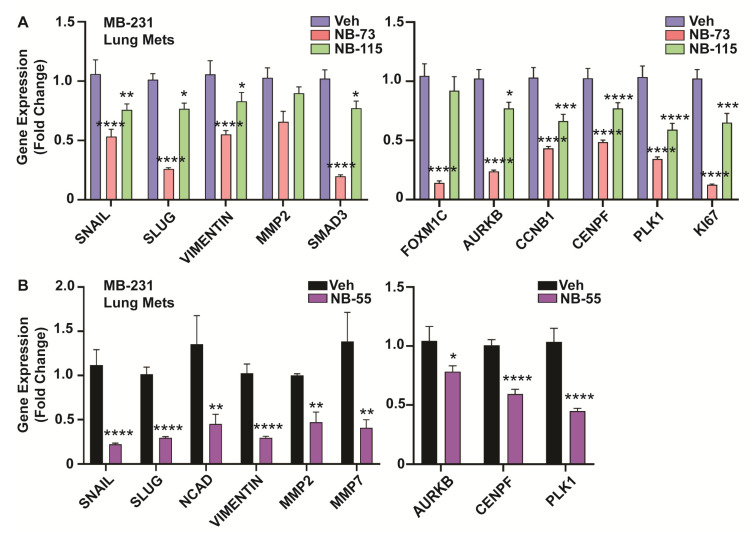
Gene expression analyses in TNBC lung metastatic lesions, Metastatic foci in the lung tissues from all animals in the compound- and vehicle-treated groups were harvested at the end of the study, followed by RNA isolation and qPCR analysis for EMT markers and known FOXM1 regulated cell cycle target genes. (**A**) Treatment with NB-73 and NB-115 or (**B**) NB-55 resulted in significant downregulation of the mRNA for different EMT markers and FOXM1 regulated genes. (2-way ANOVA, Dunnett’s post-test, * *p* < 0.05, ** *p* < 0.01, *** *p* < 0.001, **** *p* < 0.0001; values are mean ± SEM).

We examined the expression of EMT related markers in RNA isolated from primary tumors, as well as metastatic lung lesions of the control vehicle and compound treatment groups. In primary tumors, as was observed in in vitro cell studies, we saw significant downregulation of the EMT genes Snail, Slug, NCAD, Vimentin, MMPs (MMP2 and MMP7), and SMAD3, and also reduced expression of known FOXM1 target genes, such as AURKB, CENPF, PLK1, and CCNB1 in breast tumors of NB-73, NB-115 (Figure 6A) and NB-55 treated animals (Figure 6B). Similar analyses carried out on the metastatic lung lesions (Figure 7A,B) also showed reduced expression of EMT marker genes (left panels) and FOXM1 target genes (right panels) in animals treated with these compounds. Of note, NB-73 was more effective than NB-115 in suppressing these gene expressions (Figure 7A) when the compounds were administered to animals at the same dosage. NB-55, the only orally active inhibitor, was quite effective in reducing primary tumor growth and reducing the metastatic load (Figure 5D–F) and suppressing these EMT and FOXM1 target gene expressions in the lung metastatic lesions (Figure 7B).

## 3. Discussion

This study demonstrates the ability of 1,1-diarylethylene compounds to inhibit breast cancer invasive and metastatic properties in TNBC cell lines, in mouse primary tumor xenografts, and in tumor metastases that developed from these triple negative mammary tumors. These compounds suppress the activity of the cell cycle regulator FOXM1, which is overexpressed in many breast cancers, and in a variety of other cancers, including ovarian, pancreatic, lung, colon, and glioblastoma [3,5,6,10,11,20,21]. FOXM1 is especially upregulated in TNBC compared to the other breast cancer subtypes. Therefore, its role in cell cycle regulation and in transcriptional control of metastasis-related factors makes FOXM1 a unique therapeutic target in triple negative breast cancer.

In this study, we observed that inhibition of FOXM1 activity by these compounds led to decreased breast cancer cell migratory and invasiveness properties, and that this was associated with the suppression of key factors involved in the EMT process as well as other FOXM1 target genes associated with tumor progression. Furthermore, in breast tumor orthotopic studies, animals treated with these compounds, either subcutaneously (NB-73) or orally (NB-55), exhibited significantly reduced primary tumor growth and metastatic burden. NB-115 reduced primary tumor growth, but did not significantly reduce metastatic load, possibly reflecting its somewhat lower potency and efficacy in suppressing migration and invasiveness of cells in culture. It is possible that higher doses of NB-115 would have suppressed metastasis.

We examined EMT-associated factors because EMT is considered to be crucial for acquiring the loss of cell adhesion and the ability to migrate and invade. In addition to being an essential step in tumor metastasis, EMT seems to also confer stem cell-like features that contribute to treatment resistance and poor patient outcome [19,22,23]. We earlier showed FOXM1 to play a key role in promoting endocrine resistance in hormone receptor-positive breast cancers by expanding the cancer stem-like cell population [4]. While FOXM1 plays a critical role in these processes in breast cancer cells and tumors, we do not know that the effect of the NB compounds is specific to only FOXM1 inhibition in these cells.

Our new findings expand upon previous studies in other types of cancer by showing FOXM1 to be a master regulator of triple negative breast cancer progression and metastasis [16]. For example, in pancreatic cancer and glioma, FOXM1 was found to stimulate invasion and angiogenesis via regulation of MMPs [8,24,25]. In addition, gene expression profiling in metastatic prostate cancer and in TNBC showed FOXM1 to be one of the top differentially expressed genes, with the potential to regulate many of the signaling pathways that control the hallmarks of cancer [11,26]. 

Interestingly, in our study, we observed changes in expression of key players in the EMT process, including Snail, Slug, Vimentin, and MMP2, with administration of these FOXM1 inhibitory compounds. These genes are known to be under direct transcriptional control by FOXM1 in different cancers [27,28]. Our results corroborate and extend prior knowledge by showing that inhibition of FOXM1 alters metastatic properties and suppresses the progression of TNBC, and that these are associated with the downregulation of EMT factors. Because we have demonstrated previously that the three NB compounds studied here suppress cell proliferation and increase apoptosis [12], we cannot rule out that a portion of the inhibitory effects of the compounds on cell migration and invasion may be due to reductions in cell proliferation over the 24 or 48 hours of the migration and invasion assays.

The multistep process of metastatic dissemination of cancer cells involves tumor cell invasion, survival in the blood stream, extravasation, and seeding and expansion at distant sites [29,30]. In the case of breast cancer, metastatic dissemination to distant sites contributes to the majority of patient deaths [1,22,31]. Our previous work delineated a role for these FOXM1 inhibitory compounds in eliciting a G2/M block in the cell cycle and inducing apoptosis in breast cancer cells [12]. In this study, we established their ability to curb the metastatic potential of TNBC cells both in vitro and in vivo and to suppress downstream FOXM1 target genes. Approximately 60–70% of breast cancer deaths are associated with lung metastasis, particularly in the case of TNBCs [32]. MDA-MB-231 cells metastasized primarily to lungs, mirroring what is observed for most human TNBCs, and this metastatic load was reduced by treatment with NB-73 and NB-55. The NB compounds also inhibited the migration and invasion of DT28 cells and the growth of primary DT28 tumors. The effects of the compounds on tumor growth and metastasis, and on gene expressions that we have monitored, suggest that they affect both proliferation and the migration and invasion processes that underlie metastatic outgrowth. The DT28 tumors grow relatively slowly, and although they are reported to metastasize to the lungs after a long latency [33], we did not observe metastases over the 70 days in which our studies were conducted. 

Various studies have shown an important role for immune components, such as Myeloid derived suppressor cells, and secreted cytokines due to tumor-microenvironment crosstalk, in the organ-specific choice of metastatic outgrowth [34,35,36,37]. The use of immunocompromised NSG mice in our studies with human TNBC does not enable examination of the role of the intact immune system in the process of metastasis. Hence, future studies could be directed towards using these inhibitory compounds in a syngeneic model of metastatic breast cancer to investigate the role of FOXM1 in mobilizing immune cells to facilitate metastatic progression. Use of specific small molecule inhibitory compounds, such as was done here, as opposed to knocking out FOXM1 in any particular lineage of immune cells, could allow a more holistic and integrative approach in targeting all cell populations addicted to FOXM1. 

To our knowledge, this is the first study to show the efficacy of FOXM1 inhibitory compounds in impeding the metastatic outgrowth of triple negative breast tumors in animals in vivo. Previous studies have analyzed the role of other FOXM1 inhibitors, thiostrepton and FDI-6, in inhibiting certain FOXM1 functions. FDI-6, a FOXM1-specific small molecule inhibitor, prevents FOXM1-DNA interaction leading to downregulation of FOXM1 target gene expression in cells in culture [38], but FDI-6 appears to have poor pharmacokinetic properties that limit any in vivo work [12]. On the other hand, the thiazole antibiotic, thiostrepton, was found to induce cell cycle arrest and apoptosis in breast cancer cells by depletion of FOXM1. Thiostrepton was also shown to inhibit breast cancer cell migration and transforming ability [17,39]. However, these compounds have not been tested for their ability to abrogate metastasis of breast cancers in animal models. 

The FOXM1 inhibitory compounds we studied here, by curbing the EMT process, might enhance therapeutic effectiveness when combined with conventional drug treatments, including standard-of-care chemotherapies and other experimental agents. Such combination treatments may reach maximum efficacy at lower dosages, reducing the possibility of off-target drug toxicity. Overall, our findings indicate that these new FOXM1 targeting compounds may have therapeutic potential in treating TNBC with the aim of reducing tumor progression and metastatic outgrowth, and hopefully providing clinical benefit ultimately to patients with this aggressive type of breast cancer. 

## 4. Materials and Methods

### 4.1. Cell Lines and Cell Culture Methods 

The breast cancer cell line MDA-MB-231 was obtained from the American Type Culture Collection (ATCC, Manassas, VA, USA) and was maintained and cultured as described [4,12]. DT28 cells were derived from a human triple negative invasive ductal breast carcinoma and were grown in culture as described [33]. All cells were tested for mycoplasma using PCR Mycoplasma Detection Kit (Agilent, Santa Clara, CA, USA). 

### 4.2. Migration and Invasion Assays

MDA-MB-231 and DT28 cells were examined in migration assays using 8.0 µm PET membrane cell culture inserts (VWR) in a 24-well tissue culture plate. For each condition, 25,000 cells were resuspended in 200 µL serum free medium with or without the FOXM1 inhibitory compounds and seeded into the upper chamber of cell culture inserts in triplicate. The lower chambers were filled with 900 µL medium containing 10% FBS as a chemoattractant, also with or without the compounds at the same concentration as in the upper chamber. Migration was allowed to proceed for 48 h before membranes were fixed with 100% methanol and stained with crystal violet. The bottom surface of each membrane was photographed, and migrated cells were quantified using Image J software as described [4].

The classical scratch migration assay was also conducted. Cells (3 × 10^5^) were seeded in each well of a 6 well plate and grown to confluence. Then intersecting vertical and horizontal scratches were made on the cell layer using 20µL tips, followed by replacement of media with 5% serum containing either vehicle or FOXM1 inhibitory compounds. Images were captured at 24 h and 48 h post-treatment using an Evos XL Core Cell Imaging system (ThermoFisher Scientific, Waltham, MA, USA).

Invasion assays were performed using BioCoat Matrigel invasion chambers (Corning, Corning, NY, USA). Briefly, 25,000 pre-treated cells were suspended in 500 µL serum-free medium with or without treatment compounds and added to the upper chamber of inserts in triplicate. The lower chambers were filled with 750 µL of medium containing 10% FBS with or without compounds. Invasion was allowed to proceed for 48 h, after which non-invading cells were removed from the upper surface of the Matrigel-coated membrane before fixing with 100% methanol and staining with crystal violet. The bottom surface of the Matrigel-coated membrane was photographed and invaded cells were quantified using ImageJ software (National Institutes of Health, Bethesda, MD, USA) as described [4].

### 4.3. Western Blot Assays

For Western blot analysis, whole-cell extracts were prepared using 1X RIPA lysis buffer (Thermo Fisher, Waltham, MA, USA) supplemented with 1X protease inhibitor cocktail (Millipore-Sigma, Burlington, MA, USA). Proteins were separated on 4–12% SDS-PAGE gels and transferred to nitrocellulose membranes. Cell lysates were collected following treatment with compounds for the time indicated. Western blotting used antibodies against FOXM1 (Abcam, Cambridge, UK, catalog number 184637; Cell Signaling Technologies, MA, D12D5), and β-actin (Millipore-Sigma, Burlington, MA, USA, A2228) as an internal loading control. Antibodies against the EMT markers were purchased from Cell Signaling Technologies, Danvers, MA, USA (Cat# 9782) and used at dilutions recommended by the manufacturer. Both IRDye 800 CW goat anti-rabbit secondary antibody (LI-COR, Lincoln, NE, USA, Cat# 926-32211) and IRDye 680 CW goat anti-mouse secondary antibody (LI-COR, Cat# 926-68070) were diluted 1:5000 for incubation with the blots. Band intensities were analyzed with LI-COR Odyssey CLx 2.1 software.

### 4.4. RNA Isolation and Real-Time PCR

Total RNA was isolated using TRIzol (Invitrogen) and reverse transcribed using MMTV reverse transcriptase (New England BioLabs, Boston, MA, USA). Real-time PCR was performed using SYBRgreen PCR Master Mix (Quantabio, Beverly, MA, USA) as described [4]. Relative mRNA levels of genes were normalized to the housekeeping gene 36B4, and fold change calculated relative to the vehicle treated samples. Data are the average ± SEM from at least two independent experiments carried out in triplicate. Primer sequences for the genes studied were designed using Primer Blast program in the NCBI website. The sequences are listed in Table 1.

### 4.5. In Vivo Breast Cancer Xenograft Growth and Metastasis 

All experiments involving animals were conducted in accordance with National Institutes of Health (NIH, Bethesda, MD, USA) standards for the care and use of animals, with protocols approved by the University of Illinois IACUC (IACUC Protocol 20111). To study the effects of FOXM1 inhibitory compounds on breast tumor growth and metastasis, intact female NOD-SCID-gamma (NSG) mice (8 weeks of age, from Jackson Labs, Bar Harbor, ME, USA) were used as detailed previously [40]. MDA-MB-231 and DT28 luciferase expressing breast cancer cells were generated using previously described methods [41]. Cells were transfected with firefly luciferase/GFP using RediFect Red-FLuc-GFP Lentiviral Particles (Perkin Elmer, Waltham, MA, USA, CLS960003) as described by the manufacturer, and sorted for GFP fluorescence by flow cytometry with the fluorescence-activated cell sorter BDFACS AriaII. 1×10^6^ MDA-MB-231 or DT28 cells in a total volume of 100 μL were injected subcutaneously into the fourth left inguinal mammary gland of each mouse. After 2 days, mice were randomly distributed into groups (*n* = 10) to receive various treatments either by subcutaneous (s.c.) injection (NB-73 or NB-115 at 10 mg/kg mouse body weight) or by oral gavage (NB-55 at 100 mg/kg). For s.c. injection, each compound was dissolved in 100% Ethanol (EtOH) and then mixed with corn oil for a total injection volume of 100 μL (10% EtOH + 90% corn oil) per mouse. For oral gavage (OG), compounds were administered in a 200 µL formulation of 9/0.5/0.5/90 parts of PEG400/Tween80/Povidone/0.5% Carboxymethylcellulose in deionized water. In NB-73, NB-115 and s.c. vehicle groups, the mice received s.c. injection of vehicle or compound daily (5 days/week) for 2 weeks, followed by alternate day injections, three times a week for 2 weeks, and then daily injections (5 days/week) until the end of the study. For NB-55 and the corresponding Vehicle (oral gavage) group, mice were treated daily (5 days/week) to day 10, followed by alternate day injections for 2.5 weeks and then daily (5 days/week) treatment until the end of the study. Tumor volume (length × width^2^/2) was monitored over time. 

### 4.6. IVIS Bioluminescence Imaging

The extent of metastasis was followed using an IVIS Spectrum CT imaging system. Animals were injected intraperitoneally with D-luciferin (Regis Technologies, Morton Grove, IL, USA) at 150 mg/kg mouse body weight; luciferase activity was measured, and bioluminescence was quantified using Living Image software (PerkinElmer, Waltham, MA, USA) as previously described [40]. To measure the metastatic burden, the primary tumor was covered with a black box to prevent signal saturation and inaccurate measurement of metastasis, and bioluminescence was monitored.

### 4.7. Statistical Analyses

Statistics were calculated using analysis of variance (ANOVA), 2-way ANOVA with multiple comparisons, or Student’s *t*-test, as appropriate, using GraphPad Prism 8.0 software. Significance was designated as * for *p* < 0.05, ** for *p* < 0.01, *** for *p* < 0.001, and **** for *p* < 0.0001. 

### 4.8. Availability of Data and Materials

The authors declare that data supporting this study are available within the paper and its supplementary information. Other original data files and materials will be available from the corresponding author (BSK) upon reasonable request.

## 5. Conclusions

Because high FOXM1 expression is common in TNBC, we examined in this study the possibility of reducing TNBC aggressiveness and metastasis by inhibiting FOXM1 activity. We tested the ability of three FOXM1 inhibitory compounds (NB-73, NB-115, and NB-55) to suppress TNBC progression and tumor metastasis. We observed a significant decrease in motility and invasiveness of TNBC cells when treated with these compounds in cell culture, and our in vivo studies revealed that the FOXM1 inhibitory compounds attenuated tumor growth and distant metastasis. These effects were accompanied by reductions in the level of FOXM1 target genes and important players in epithelial to mesenchymal transition (EMT), including Snail, Slug, Vimentin, and MMP2. To our knowledge, this is the first study to show the efficacy of FOXM1 inhibitory compounds in impeding the metastatic outgrowth of triple negative breast tumors in animals in vivo. These observations highlight the crucial role of FOXM1 in promoting the progression and metastasis of TNBC, and suggest that these compounds may hold promise for therapeutic intervention against TNBC, with the aim of reducing tumor progression and metastatic outgrowth, and hopefully might ultimately provide clinical benefit to patients with this aggressive type of breast cancer.

## Figures and Tables

**Table 1:** Sequences of primers used in this study.

Gene	Forward Primer	Reverse Primer
*Snail*	5′-TAGCGAGTGGTTCTTCTGCG-3′	5′-GTTAGGCTTCCGATTGGGGT-3′
*Slug*	5′-GAGCATACAGCCCCATCACT-3′	5′-CTCACTCGCCCCAAAGATGA-3′
*Vimentin*	5′-AAACTTAGGGGCGCTCTTGT-3′	5′-CGCTGCTAGTTCTCAGTGCT-3′
*Ncad*	5′-GAGGCTTCTGGTGAAATCGC-3′	5′-AGAAGAGGCTGTCCTTCATGC-3′
*MMP2*	5′ATCCAGACTTCCTCAGGCGG-3′	5′-TCCTGGCAATCCCTTTGTATGT-3′
*SMAD3*	5′-CCATCTCCTACTACGAGCTGAA-3′	5′-CACTGCTGCATTCCTGTTGAC-3′
*FoxM1*	5′-CAATTGCCCGAGCAGTTGGAATCA-3′	5′- TCCTCAGCTAGCAGCACCTTG-3′
*36B4*	5′-AGCCCAGAACACTGGTCT-3′	5′-ACTCAGGATTTCAATGGTGCC-3′
*AurkB*	5′-ACGATCATGGAGGAGTTGGC-3′	5′-CCCTTGAGCCCTAAGAGCAGA-3′
*Ccnb1*	5′-CGGGAAGTCACTGGAAACAT-3′	5′-AAACATGGCAGTGACACCAA-3′
*Cenpf*	5′-CTCTCCCGTCAACAGCGTTC-3′	5′-GTTGTGCATATTCTTGGCTTGC-3′
*Plk1*	5′-AAAGAGATCCCGGAGGTCCTA-3′	5′-GGCTGCGGTGAATGGATATTTC-3′
*Ki67*	5′-ACGCCTGGTTACTATCAAAAGG-3′	5′-CAGACCCATTTACTTGTGTTGGA-3′

## Supplementary Materials

The following are available online at https://www.mdpi.com/2072-6694/12/9/2677/s1, Table S1: Antibodies used in these studies; Figure S1: Effect of FOXM1 inhibitors on motility of MDA-MB-231 cells evaluated by Scratch Assay and effects on FOXM1 target gene expression; Figure S2: Body weights of vehicle and compound treated animals during the time course of tumor studies with MDA-MB-231 and DT28 cells; Figure S3: (A) Full Western Blots for the proteins shown in Figure 4A (left), (B) Full Western Blots for the proteins shown in Figure 4A (right); Figure S4: (A) Full Western Blots for the proteins shown in Figure 4B (left), (B) Full Western Blots for the proteins shown in Figure 4B (right).
