# Supplementary material for: Suppression of Tumor Growth, Metastasis, and Signaling Pathways by Reducing FOXM1 Activity in Triple Negative Breast Cancer
Source: Cancers (Basel). 2020 Sep 19;12(9):2677. doi: 10.3390/cancers12092677 (PMC7565743; doi:10.3390/cancers12092677)
Supplement: Supplementary file 1 [file cancers-12-02677-s001.pdf]

Supplementary Materials: Suppression of Tumor Growth, Metastasis, and Signaling Pathways by Reducing FOXM1 Activity in Triple Negative Breast Cancer

Parama Dey, Alexander Wang, Yvonne Ziegler, Sung Hoon Kim, Dorraya El-Ashry, John A. Katzenellenbogen and Benita S. Katzenellenbogen

Supplementary Table S1 – Antibodies used in these studies

**Antibody**	**Source**	**Dilution**
FOXM1	CST D12D5	1:500
SNAIL	CST (C15D3)	1:750
SLUG	CST (C19G7)	1:750
VIMENTIN	CST (D21H3)	1:1000
ACTIN	Sigma (A2228)	1:5000
MMP2	AbCam (ab37150)	1:1000

Supplementary Figure S1

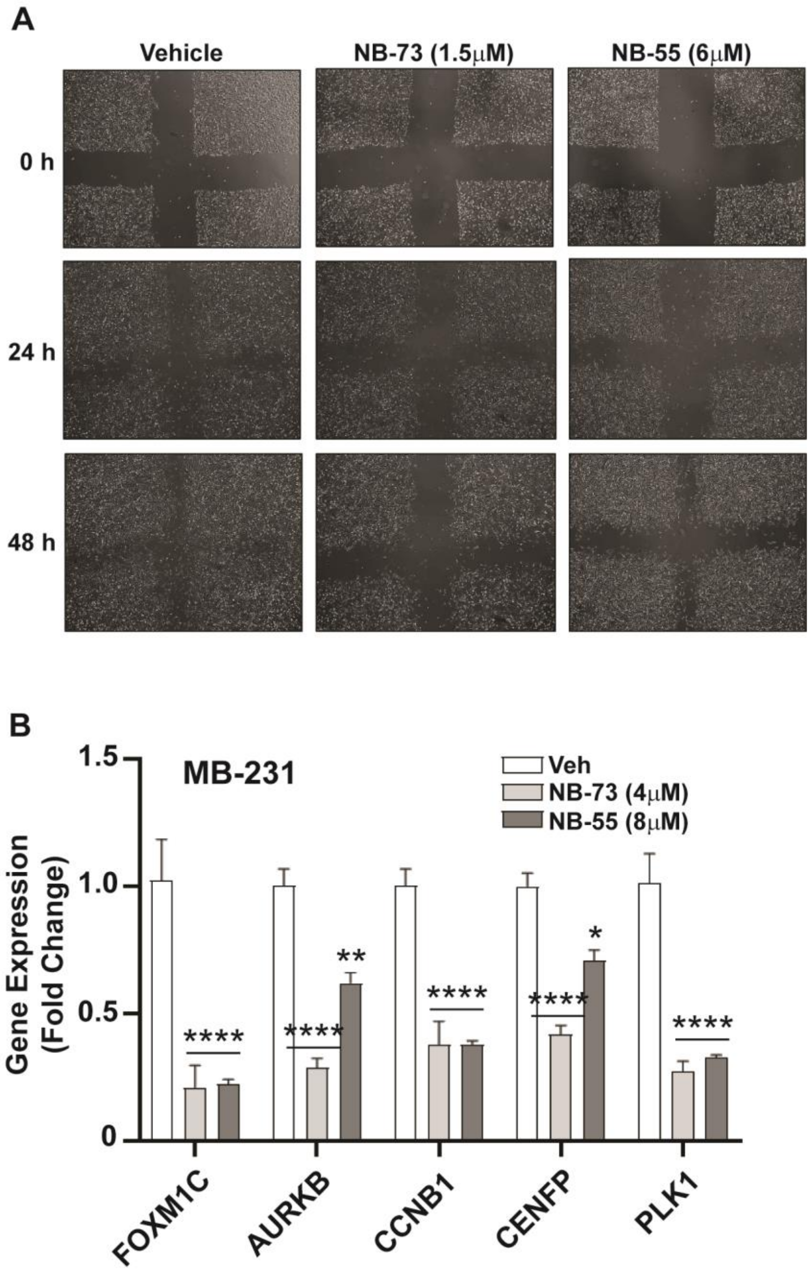

Fig. S1. Effect of FOXM1 inhibitors on motility of MDA-MB-231 cells evaluated by Scratch Assay and effects on FOXM1 target gene expression

(**A**) Cells (3x10^5 MDA-MB-231 cells) were seeded in each well of a 6 well plate and grown to confluence. Then intersecting vertical and horizontal scratches were made on the cell layer using 20μl tips followed by replacement of media with 5% serum containing either vehicle or FOXM1 inhibitory compounds. Images were captured at 0 h, 24 h, and 48 h post-treatment using Evos XL Core Cell Imaging system at 4X magnification. (**B**) MB-231 cells were treated for 24 h with 4μM NB-73 or 8μM NB-55 followed by RNA extraction and q-PCR analysis of FOXM1 and FOXM1 target gene expressions. Statistical analysis used 2-way ANOVA and *p<0.05, **p<0.01, ***p<0.001, and ****p<0.0001 are indicated; mean ± SEM.

Supplementary Figure S2

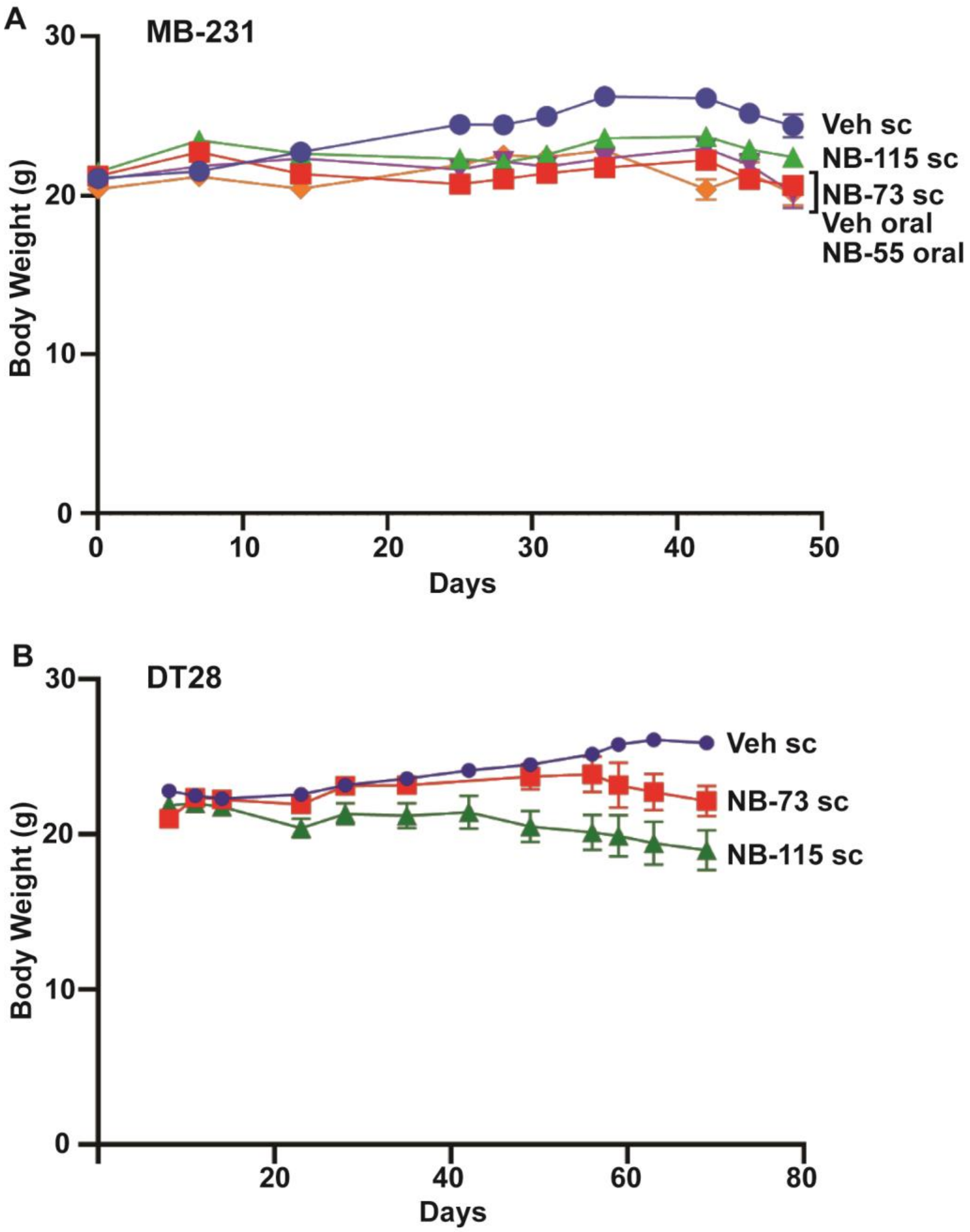

Fig. S2. Body weights of vehicle and compound treated animals during the time course of tumor studies with MDA-MB-231 and DT28 cells.

Body weights of NSG mice carrying (**A**) MB-231 or (**B**) DT28 tumors were measured biweekly during treatments sc with vehicle or NB-73 and NB-115, or by oral gavage with vehicle or NB-55. Values are mean ± SEM. Error bars are not visible for some points because they are smaller than the point symbol. Note that the x-axis scale (time in days) is different in Panels A and B.

Supplementary Figure 3A

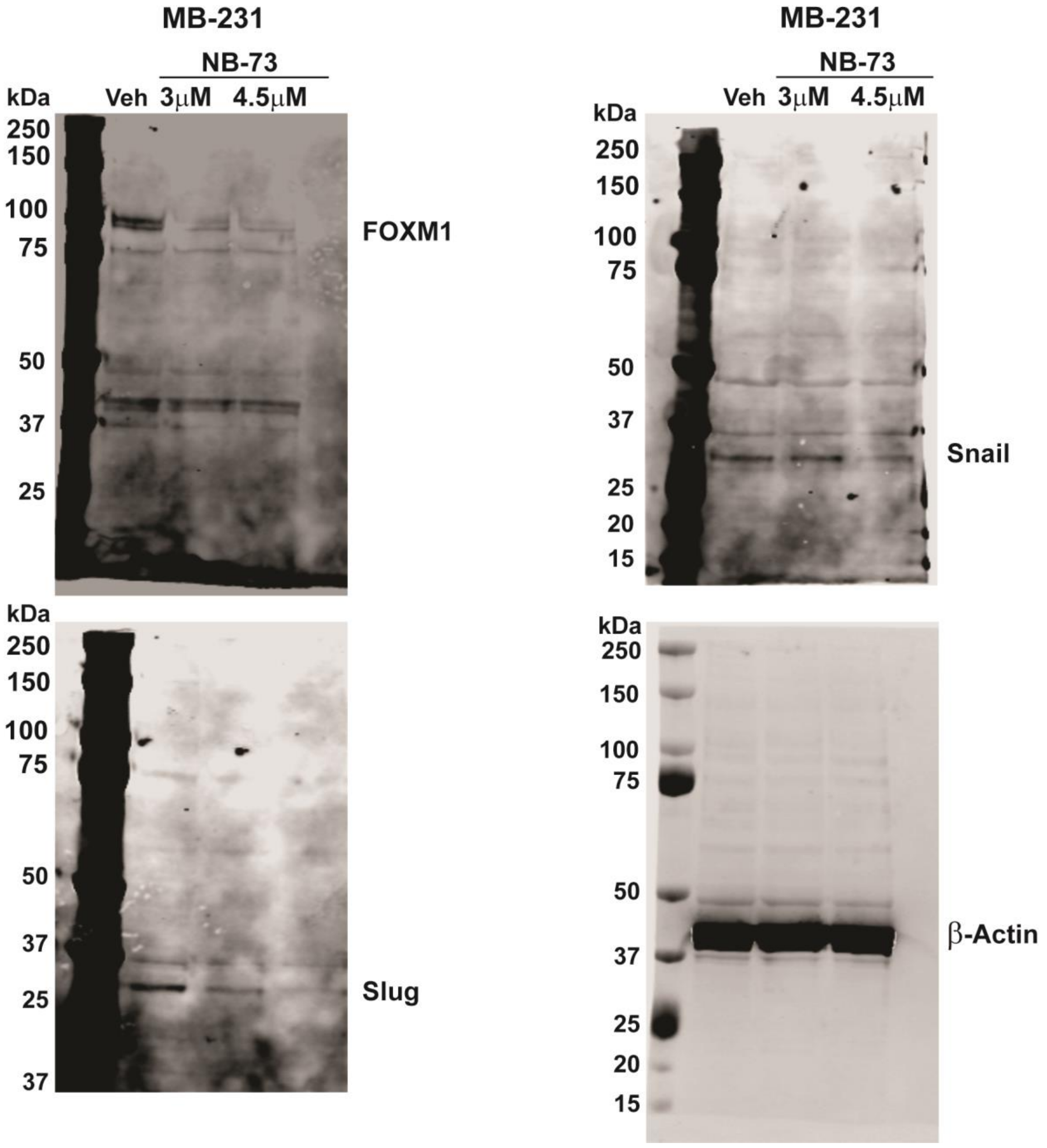

Fig. S3A. Full Western Blots for the proteins shown in Figure 4A (left). MB-231 cells were treated with vehicle or compound NB-73 at the concentrations indicated and blots were probed with antibody for the protein indicated. Molecular weight markers are shown in the left-most lane with their kDa noted.

Supplementary Figure 3B

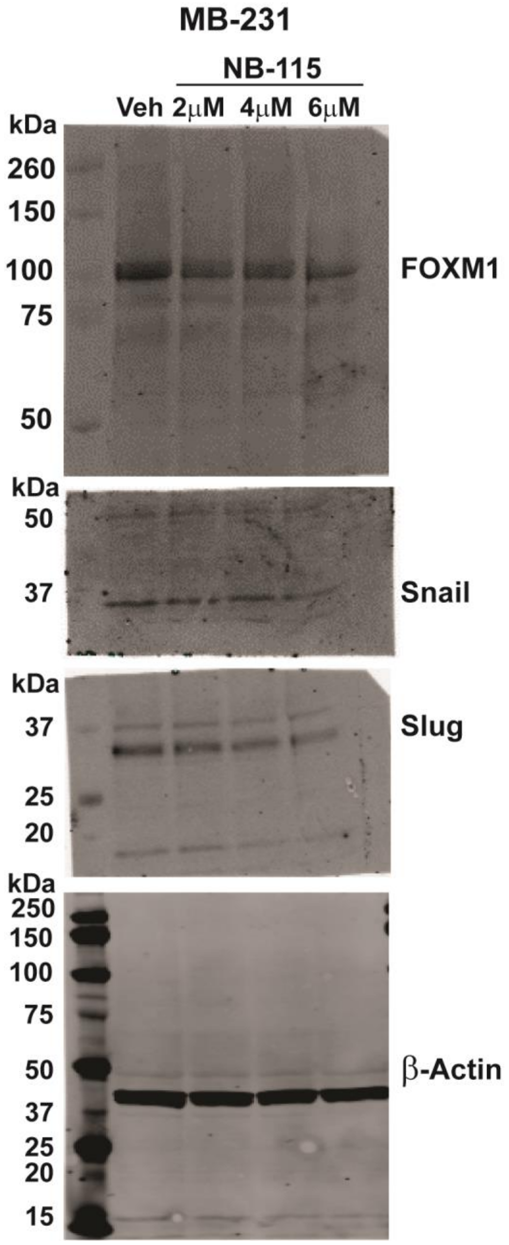

Fig. S3B. Full Western Blots for the proteins shown in Figure 4A (right). MB-231 cells were treated with vehicle or compound NB-115 at the concentrations indicated and blots were probed with antibody for the protein indicated. Molecular weight markers are shown in the left-most lane with their kDa noted.

Supplementary Figure 4A

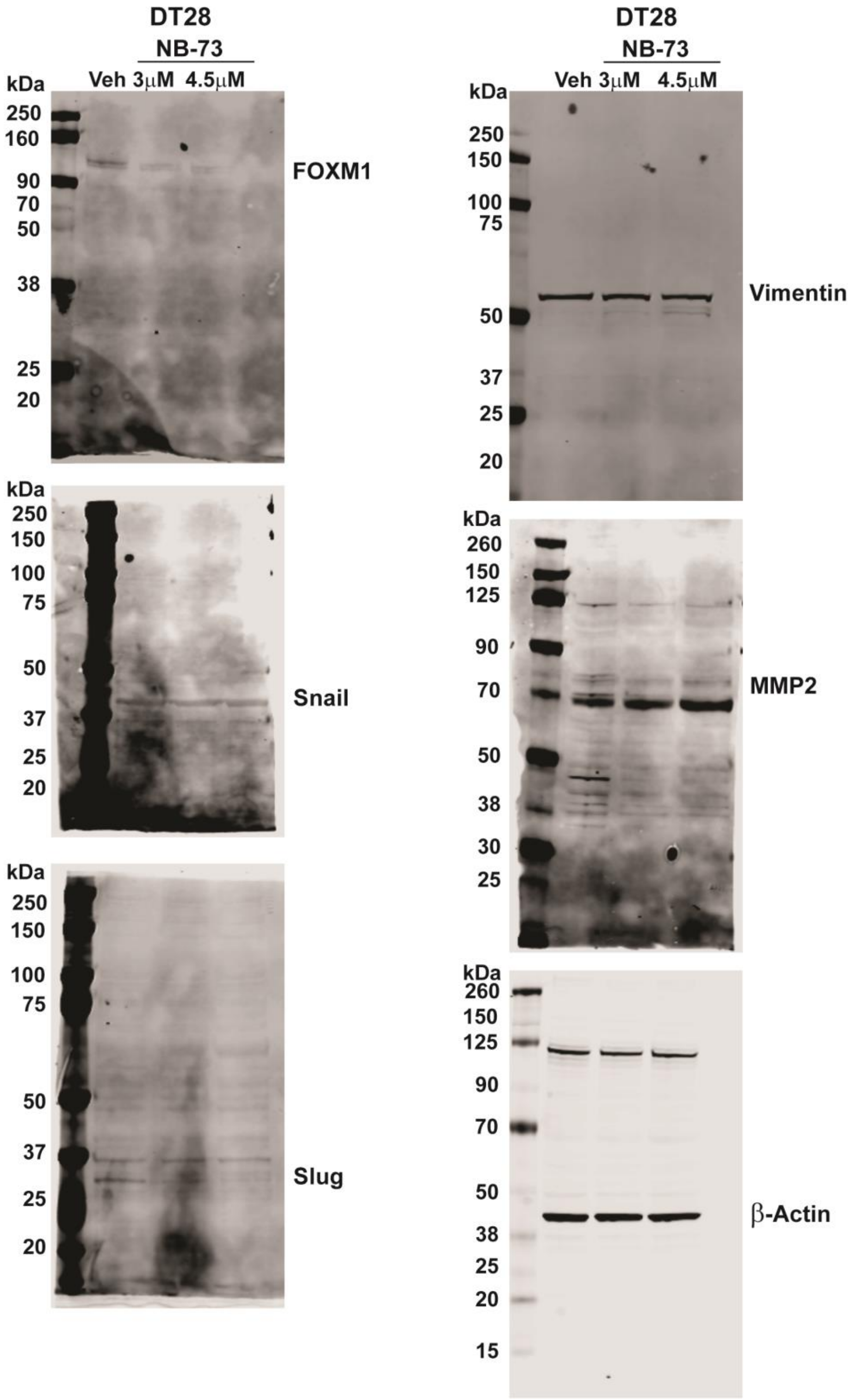

Fig. S4A. Full Western Blots for the proteins shown in Figure 4B (left). DT28 cells were treated with vehicle or compound NB-73 at the concentrations indicated and blots were probed with antibody for the protein indicated. Molecular weight markers are shown in the left-most lane with their kDa noted.

Supplementary Figure S4B

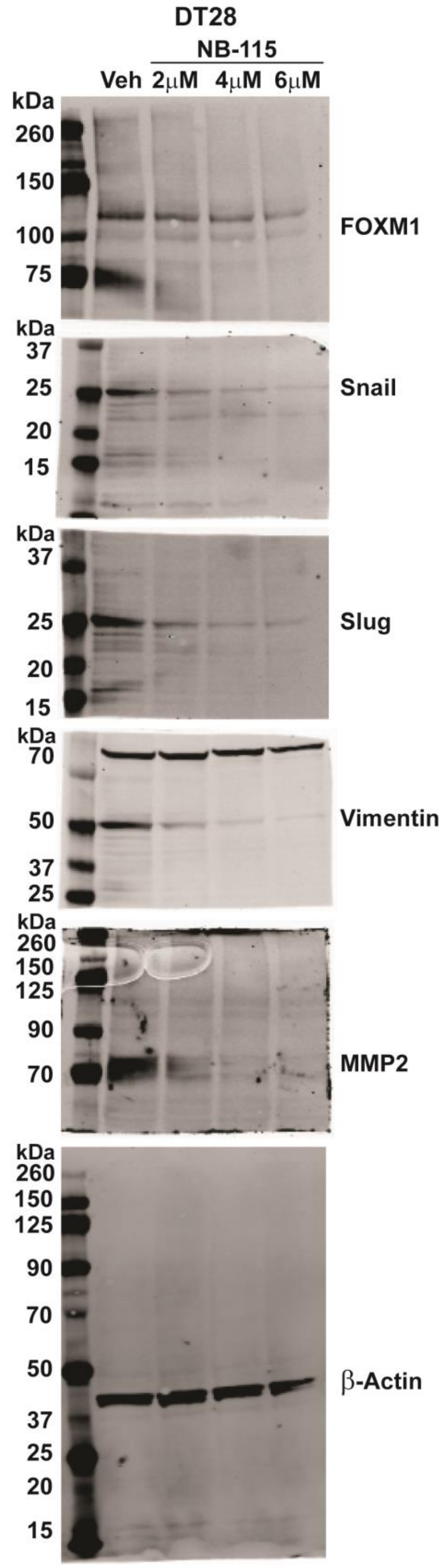

Fig. S4B. Full Western Blots for the proteins shown in Figure 4B (right). DT28 cells were treated with vehicle or compound NB-115 at the concentrations indicated and blots were probed with antibody for the protein indicated. Molecular weight markers are shown in the left-most lane with their kDa noted.
